# A phenomenological analysis of the experience of taking medication to prevent a further heart attack

**DOI:** 10.1038/s41598-021-02909-5

**Published:** 2021-12-06

**Authors:** Hannah Piekarz, Catherine Langran, Parastou Donyai

**Affiliations:** School of Pharmacy, University of Reading, Harry Nursten Building, Whiteknights, Reading, Berkshire RG6 6DZ UK

**Keywords:** Cardiology, Health care, Diseases, Cardiovascular diseases

## Abstract

Following an acute myocardial infarction, patients are prescribed a regime of cardio-protective medication to prevent recurrent cardiovascular events and mortality. Adherence to medication is poor in this patient group, and not fully understood. Current interventions have made limited improvements but are based upon presumed principles. To describe the phenomenon of medicine-taking for an individual taking medication for secondary prevention for an AMI, Interpretative Phenomenological Analysis was used to analyse transcripts of semi-structured interviews with participants. Themes were generated for each participant, then summarized across participants. Five key themes were produced; the participants needed to compare themselves to others, showed that knowledge of their medicines was important to them, discussed how the future was an unknown entity for them, had assimilated their medicines into their lives, and expressed how an upset to their routine reduced their ability to take medication. Participants described complex factors and personal adaptations to taking their medication. This suggests that a patient-centred approach is appropriate for adherence work, and these themes could inform clinical practice to better support patients in their medicine adherence.

## Introduction

Cardiovascular disease currently causes over 4 million deaths annually in Europe^1^. Acute myocardial infarction (AMI) is responsible for the largest proportion of these, estimated to be 15% of the total^2^. In the UK, just under one million people are thought to be AMI survivors^3^.

Following an AMI, a combination of five different classes of medicines are recommended as part of treatment guidelines for secondary prevention in the UK^4^, the US^5^, and Europe^6^. A meta-analysis of these drug groups has found that following the five-drug therapy confers a 40% reduction in mortality and 25% reduction in cardiovascular events^7^. Whilst a broader study in coronary heart disease patients, it found that the reduction in all-cause mortality and cardiovascular events was due to an additive effect. This highlights the importance of taking these drugs in accordance with the prescription issued by a health professional.

Medicine adherence is defined as “the extent to which a patient’s behaviour matches agreed recommendations from the prescriber”^8^. Meta-analytic data from 376,162 patients has shown the level of adherence in patients taking medicines for secondary prevention of AMI to be 66%^9^. This study found no other statistically significant differences between the drug classes, which suggests that non-adherence is not related to a drug class characteristic, such as a drug side-effect, but to other factors. Because non-adherence in AMI increases one-year mortality, hospitalisations and costs^10^, it follows that improving medicine adherence should then reduce patient mortality, morbidity and healthcare system costs.

Improving medicine adherence has been a focus for the World Health Organisation (WHO)^11^, which famously reported that “increasing the effectiveness of adherence interventions may have a far greater impact on the health of the population than any improvement in specific medical treatments”.

In the UK, the standards of practice for the NHS are determined by the National Institute for Health and Care Excellence (NICE), which has produced treatment guidelines for improving medicine adherence^12^. This report acknowledges the need to understand patient perceptions of their medicine along with the physical practical support that can be given to aid patient adherence. It recommends that adherence is supported through patient involvement in decision-making, offering information, and being aware of patient concerns.

There has been much research into finding effective interventions, yet many are complicated and eventually unsuccessful in terms of improving medicine adherence and clinical outcomes^13^. Most adherence research uses quantitative methods to determine adherence as an outcome, with a thin evidence base underpinning the theoretical framework. A study of the data from adherence studies concluded that most interventions are not produced as a result of theoretical models^14^, even though interventions aimed at changing behaviour have been shown to be more effective when based upon theoretical models^15^. Therefore, a qualitative approach would be useful to generate themes and propose models which could ratify conceptual frameworks and direct further work in quantitative studies. Historically, there is a lack of qualitative research into medicine adherence^16^.

Consistent with a patient-centred approach, a qualitative phenomenological study is appropriate to obtain a patient perspective of the experience of taking medicines, beliefs, and how patients conceptualise their medicines within their lives^17^. In addition, a study that includes an exploration of the social context and lived world of a patient would be appropriate, and an ethnographic patient interview is suited to this purpose^18^.

Interpretative phenomenological analysis (IPA) was chosen as an established accessible methodological framework upon which to base this study^19^. There is debate between the leading practitioners in phenomenology as to the philosophical underpinnings of their own respective methods^20^. In order to develop a deeper phenomenological aspect to this study, the additional framework of existential life-world categories of time, location, embodiment and relationships were used in the analysis^21^.

### Aim of the study

To describe the experience of an individual taking medication to prevent a further AMI, and factors that influence their medicine-taking ability.

### Ethics approval

This study was approved by the University of Reading Ethics Committee (Reference UREC18/36).

## Method

This study followed COREQ guidelines to conduct the research^22^. A sample size of four participants was chosen to enable a quality analysis to take place, to ensure thoroughness, depth and maintain ideography of the analysis, consistent with an Interpretative Phenomenological Analysis (IPA) study^23^.

Participants were recruited using a promotional poster cascaded by email within the University of Reading, which was also sent to targeted cardiac support groups within the locality of the South-East region of the UK. The criteria for inclusion were adult individuals with a diagnosis of AMI and who had been prescribed medication for secondary prevention of AMI.

Initial contact was made through email with the first author, HP, the study information was distributed, and written consent returned in person or via email. Prior to interview, participants were assigned a pseudonym. All participants who made initial contact followed through to full interview.

All interviews were conducted by HP in a private office room, either face to face or over the telephone, between June 2019 and January 2020. The interviews ranged in duration from 17 to 65 min, and average time of 48 min.

A semi-structured interview schedule was used to guide the interviews. The schedule was informed by one used in a similar medicine adherence phenomenological study^24^, and more general literature on developing interview protocol^25^. Following the first interview, the schedule was refined in accordance with a self-reflective ‘interview the interviewer’ technique^26^, the final schedule can be found in Additional Information. All listed questions were put to the participants, and during the interview, further lines of questioning and probes were added in response to answers given by the participant as the interview progressed.

Field notes were taken by HP during the interviews as an aid to topic coverage. Immediately following the interview, notes were made on meaning of discussion topics, to aid in the analysis. The notebook was used during the transcription and analysis stages also, to add a reflexive commentary on the researcher’s thoughts and sense-making process.

Interviews were audio-recorded to MP3 file, which was transcribed verbatim by HP into an MS Word text document. The transcripts were checked alongside the audio to confirm accuracy. In line with a transparent study, participants were sent a copy of their transcript. No participants disagreed with the content of their transcript.

Analysis was undertaken according to IPA^23^. All transcripts were analysed by HP, a novice qualitative researcher, PhD student and practicing pharmacist. The transcripts were read a minimum of twice, and line-by-line notes made using an IPA stance, a description of the meaning and understanding by the researcher of the participant’s statements. The first transcript notes were discussed with PD, an experienced qualitative researcher and Professor of pharmacy practice, with specialisms in anthropology and psychology. This discussion generated further notes, consistent with a “mini-independent audit”^23^, a check of validity. This discussion of notes was repeated for the second transcript.

Life-world framework categories of time, location, embodiment and relationships were used as an initial means of organising and arranging the notes^21^. The transcript notes were colour-highlighted by life-world category and grouped.

Following the first sift into lifeworld groups, the transcript notes were coded into thematic groups, collected together according to concept. These groups were used to write a participant summary document in prose, which described each individual, arranged by life-world category. In line with IPA, preservation of the participant’s voice is important, and so the original interview quotes were used to illustrate themes. Analysis was repeated for each transcript and a summary document was produced for each participant.

The summaries of themes for each participant were grouped together, using overarching themes that encompassed all the concepts encoded in the themes that they described. These were the superordinate themes that featured across all the participants’ transcripts. These superordinate themes were organised into tables illustrated with the original text quotes from each participant which are presented in the results section below.

## Results

The participants (n = 4, 2 women, 2 men) were assigned the pseudonyms Gaye (Table 1), Beki (Table 2), Chris (Table 3), and Colin (Table 4). They all described their AMI as a “heart attack” and so this term is used henceforth. All participants lived in the South-East region of the UK and came from higher professional or business-owner backgrounds in their working career. They all initially received emergency care through the NHS and continued their access to healthcare through their NHS general practitioner service. Beki and Chris sought additional care through using private consultant cardiologists.

**Table 1:** Summarised themes and example interview quotes from Gaye.

Superordinate theme	Theme	Quote	Transcript line reference
Comparison to others	Health conscious	Lifelong vegetarian	44
		Never taken medication	65
		…Because I was living that lifestyle (Re: survival)	152
		As fit as a butcher’s dog	169
	Unusual case	Didn't fit the bill for anyone having a heart attack	43
		Didn’t fit anyone’s profile	48
Knowledge	Medicine information	[Medicine names, dosage and times]	77, 79
	Question cause	Why had it happened,	146
	Question medicines	Are they doing what they’re supposed to?	219
		What would happen if I didn’t take them?	220
		How does the combination work?	234
	Self-perception (medicines)	Am I on the highest? Am I on the lowest?	224
	Self-perception (fitness)	No idea of what level you’re at	176
	Sharing knowledge	I told her what I'd been told	187
	Strength in numbers	Collective support (re: rehab group)	234
Considering the future	Planning into the future	To be seen in a couple of years	172
	Indefinite	Continue to have to take these	241
	Continuous future	Forever and being compliant	242
	Unknown future	Not knowing where I was heading…	152
Assimilation into lifestyle	Overwhelmed initially	Started off with a whole raft of things	52
	Physical issue	Could never swallow medicines	92
	Overcome issue	Always have to have grapes	113
	Taking medicine is unremarkable	It’s just a routine now	107
Disturbance to routine	Being away from home	Can be a bit difficult when you’re travelling	113
	Distracted by activity	Been a particularly busy day, it’s not until the evening when I thought, ‘Crickey!’	109

**Table 2:** Summarised themes and example interview quotes from Beki.

Superordinate theme	Theme	Quote	Transcript line reference
Comparison to others	Health conscious	Training for the marathon	5
		Planning on starting a family	137
		Never smoked	259
		Been this weight since…a teenager	558
	Unusual case	I’m not kind of, your normal case	628
		The youngest person on the cardiac ward	607
		I’m not your fat sixty or seventy year-old man	95
		My heart function is normal	456
		My heart has no damage to it	458
	Dislike of labelling	I hate that term (re: saying I have heart disease)	448
Knowledge	Medicines information	[Medicine names, dosages, timings]	77, 78,79, 159, 190, 192, 222, 246, 248
	Self-perception (medical)	Cholesterol…high for me	256
		All of the data	116
	Seeking information	Load of Googling	422
		A bit of PubMed searching	656
		A UK Facebook group	427
		A lot of my friends are Doctors	696
		Spoke to a friend… endocrinologist	657
		Sister-in-law is a consultant	734
	Cynical of care	They were generally a bit rubbish	99
		The ECG technician will answer your question	105
		Here's a bag of stuff	293
		A whole leaflet of aftercare	296
		The woman that hands me… probably has no clue	976,977
Considering the future	Future unknown	Try not to dwell, may happen again	461,462
	Continuous future	Every day for the rest of my life (re: taking medicine)	344
	Planning ahead	Lipids done every year	964
		Making an appointment	622
		Four medicines every six weeks	648
Assimilation into lifestyle	Consistent location	They're all in the kitchen	477
		Down in the kitchen	375
	Reason for location	Out of the way	478
		Aspirin has to go in water	374
	Child is priority	Had stopped taking the (contraceptive) pill	156
		Came off statin, clopidogrel	177
		Breastfed him	186
		A break while I was pregnant	971
	Concern for partner’s anxiety	Look like a battered wife (re: bruising as side effect)	902
		My poor husband…	141
		woke up, thought I was dead (re: coldness as side effect)	142
		I think my husband worries about it more than I do	442
	Side effect tolerated	For the sake of a year, it’s fine (re: clopidogrel causing bruising)	921
	Side effect unacceptable	Felt like wading through treacle	138
Disturbance to routine	Being away from home	I think probably I was out and thought, ‘oh yeah’	492, 494
	Distracted by activity	Just didn’t ’cause I came home and went straight up to bed	495

**Table 3:** Summarised themes and example interview quotes from Chris.

Superordinate theme	Theme	Quote	Transcript line reference
Comparison to others	Parallel self	That was the heart attack that would have been fatal	52
		And er, if we hadn’t gone – who knows?	85
		Thinking—I've got cancer	523
	Comparison to an acquaintance	You know, just passed away on the spot	66
	Comparison to relatives	I have two brothers. Both younger	942
		He had a triple	944
		He needed a bypass, but because of his head (re: other brother has pacemaker)	949
		We do compare notes between us	968
	Unusual case	My heart was undamaged	926
		My heart had found ways of getting supplies from other channels	924
		It was a re-plumbing job—it wasn’t a heart repair job, which I think was quite significant	936, 937, 938
Knowledge	Medicines information	[Names, doses, times and identification of medicines]	169, 182, 183, 194, 195, 196, 223,273, 284,285, 286,474
	Theoretical knowledge	And also one used for epileptics	180
		Which is again, a drug normally used for Parkinson’s disease	190
	Practical knowledge	Intelligently, I have, you know (re: self-adjusts dose)	558
		Self-diagnosed, self-prescribed	500
		Did off my own back	563
	Follows advice of professionals	I’ll stick with that one	581
		Wouldn't stop taking that one without taking advice	582
		‘Cause it helps with the heart rate	579
		If that’s what they say,	976
		I won’t question	979
	Self-perception (Medicine)	I’m on about as low as you can get	575
	Self-perception (Medical)	My readings seem to come out OK	218
Assimilation into lifestyle	Continuing as usual	And I’m taking it when it fits in with my life	205
		Just throw in the morning ‘cos it’s more convenient to me	217
		Take them when it fits me	227
	Adapting to change in routine	And I get on with it ‘cos it’s only short-term (re: antibiotic course)	340
		I’d probably do one of those for the flight time (re: small pill box)	357
		It lived in the car all day…Wanted to make sure I had the afternoon ropinirole with me	368,370
	Taking medicines is unremarkable	Sort of, pick them up, chuck them in and swallow them	297
		And taken without thinking	113
		It’s whatever’s in front of me	292
		Like having a cup of coffee or a glass of wine (analogy: routine)	535
		Coffee for that, wine for this (analogy: cultural)	1092
	Location of medicine	This square box with a green lid that’s got everything in	361
		Sometimes it's in the kitchen	364
		Sometimes the worktop, sometimes the cupboard	365
	Constructed relationship with regular medicines	My normal, you know, cocktail	343
		A routine, daily medicines, wall to wall	1023
		Doesn’t become a part of the family of the rest in my box (re: antibiotics)	336,337
		It's a bit of an intruder	339
	Made own system	Works absolutely brilliantly	361
		That's my method	389
		Sorry, but that’s the way I do it	391
	Concern for efficiency	A tenth of the time it would the other way (re: decanting medicine)	433
		It’s very timesaving	434
		An absolute disgrace (re: overpackaging)	415
	Unconcern with adhering to doses	Wouldn't have bothered me	500
		None are things that are life threatening if I miss a day or take too many	464
		Totally relaxed	467
		There’s no hassle or stress or anything, on it	478
Disturbance to routine	Distracted by activity	Something distracted me and I was doing something unusual late last night,	483
		I went to bed and forgot that I'd not taken my pills	497

**Table 4:** Summarised themes and example interview quotes from Colin.

Superordinate theme	Theme	Quote	Line reference
Comparison to others	Chance affects health	I was pretty unlucky to have a clot	469
		I’m just so lucky really	729
Knowledge	Medicine information	[Medicine names, doses, timing]	26, 28, 29, 30, 31, 32
	Seeking superior healthcare	That’s when we decided to see the consultant privately	181
		For longer than most people would recommend	76, 77
		Said ‘stay on the clopidogrel for a further year’	97
		Even though the reports say that its rubbish	218
		He reckons that it’s worthwhile	219
		This consultant, who I trust, said take them	390
		He’s pretty well-regarded in his profession	653
	Seeking information	Read the leaflet about what they do and what the side effects are	629
	Self-perception (medical)	Always was fine and always is fine	147
		Stable ever since	161
		Been settled for quite a long time	202
		Not in the last, probably nine years	505
		Been taking them for such a long time	338
		Drug has built up inside me	340
	Self-perception (fitness)	I managed a good peak time	64
		A very good walking time	153
	Financial exchange to access healthcare	The National Health wouldn’t provide them	83
		BUPA don’t think it’s entirely necessary	137
		Certainly our GP, they won’t pay for that. That's up to me	138
		Surgery stopped paying for Omacor, it was do it yourself, if you want it	141
		I can afford it, let's put it that way	452
Considering the future	Continuous future	I just have to take these forever	169
Assimilation into lifestyle	Initial difficulty	It was all so new then	22
		They just said you’re on these, and go	178
		Wasn’t in the best for a couple of days	136
		A bit difficult to know what each one was doing	37
		Felt disappointed that I was going to take medication	172
	Taking medicine is unremarkable	Just as routine as having a cup of tea	379
		Just take them and get on and do things	391
		It takes 30 s, what's the problem?	404
	Unconcern with missed doses	I don’t notice that I’ve taken anything	209
		I don’t get worried if I don’t take them	366
		I don’t notice on a daily basis	497
		I miss one then never notice any difference	501
	Location of medicines	In a little pouch, there the whole time	293,294
		Leave them out, just to remind me	290
		Take upstairs, to be by my case	607,608
		Take out the next morning’s medication	610
		The evening medication, and put cling film over that	612
		Will always have that in my pocket (re: GTN spray)	973
		Whereas I’ll clear everything else out of my pocket	978
Disturbance to routine	Distracted by activity	When all the family were here	312
		The routine is a bit different (re: holiday)	591
	Change of timings	Timing dosages to that of the place we are going (re: time zone change)	327
		Might be wider or less	329

Gaye is a retired woman, in the age range 60–70 years. She had two heart attacks and considers that her healthy vegetarian lifestyle is the reason that she survived them. She had difficulty swallowing tablets but overcame this by taking her medicines simultaneously with swallowing a grape.

Beki is a working woman in the age range 30–40 years. She had two heart attacks but considers herself an anomaly due to her fitness and young age. Her initial diagnosis was not of a heart attack, it was through her own research that she found a consultant to confirm a diagnosis of heart attack.

Chris is a retired man in the age range 60–70 years. He had one heart attack. He decants all his medication into a Tupperware box in order to save time and hassle. He also takes medication for back pain and restless leg syndrome, for which he adjusts doses according to the severity of his symptoms.

Colin is a retired man in the age range 60–70 years. He had one heart attack, whilst on the golf course and was air-lifted to hospital away from home. Dissatisfied with the quality of transfer of his care between hospital and GP, he sought private healthcare.

The participants discussed medicine-taking through four superordinate themes (Table 5); they *compared themselves* to others, *knowledge* was important to them, they considered the *future* in some form, and they discussed ways that they fitted their *medicines into their lifestyle*. A related theme to lifestyle, all participants discussed how *a change to their routine* adversely affected their medicine-taking.

**Table 5:** Summary of themes as mentioned by participant.

Superordinate theme	Subtheme	Mentioned by participant			
		Gaye	Beki	Chris	Colin
Comparison to others	Health conscious	•	•		
	Unusual case	•	•	•	
	Parallel self			•	
	Compare to acquaintance			•	
	Compare to relatives			•	
	Chance affects health				•
	Dislike of being labelled with diagnosis		•		
Knowledge	Medicines information	•	•	•	•
	Question cause	•	•		
	Question medicines	•	•		•
	Self-perception (medicines)	•		•	
	Self-perception (fitness)	•			•
	Self-perception (medical)			•	•
	Cynical of care		•		
	Overcome medicine issue	•		•	
	Seeking information		•		•
	Theoretical knowledge		•	•	
	Practical knowledge			•	
	Follows professional advice		•		•
	Seeking superior healthcare				•
	Paying for healthcare				•
	Sharing knowledge	•			
	Strength in number	•			
Considering the future	Planning ahead	•	•		
	Indefinite		•		
	Continuous future				•
	Unknown future	•	•		
Assimilation into lifestyle	Initial overwhelmed	•			•
	Concern for timing				•
	Continue as usual			•	•
	Adapting to incorporate change	•		•	
	Taking medicine is unremarkable	•		•	•
	Unconcerned with adherence			•	•
	Child is priority		•		
	Concern for partner’s anxiety		•		
	Made own system			•	
	Concern for efficiency			•	
	Constructed relationship with regular medicines			•	
	Physical issue	•			
	Side effect tolerated		•		
	Side effect unacceptable		•		
	Location of medicine		•	•	•
Disturbance to routine	Being out of the home	•	•	•	•
	Being on holiday				•
	Distracted by activity		•	•	•
	Changing of timings				•

### Comparison to others

One of the superordinate themes was the way in which participants focussed on their history and lifestyle leading up to their heart attack in reference to other people, often evidencing that they were in a superior position, and as proof that their body was healthier by managing to survive the heart attack.

This theme was interpreted as participants constructed a ‘typical’ heart attack patient profile for comparison in terms of healthy living status, gender, age and type of heart attack. For example, terms like “Didn't fit the bill for anyone having a heart attack”, and “I’m not your fat sixty or seventy year-old man”.

They frequently gave examples of how they were health conscious. They considered that vegetarianism, non-smoking, training for a marathon, preparing for pregnancy, and gym attendance to be attributes that made their health superior. They used their own biometric results to compare their health to normal ranges as justification that their health is comparable to ‘normal’. They also described similar diagnoses in acquaintances, friends and relatives to discuss how their case was different, with the implication that their case was less morbid. Colin defined himself in terms of luck.

### Knowledge

The superordinate theme of knowledge encompassed a range of themes that included questioning the cause of their heart attack and need of their medicines, seeking information, having a theoretical and practical knowledge of their medicines, sharing information with others and receiving support as part of a group. The category of knowledge also encompassed self-perception, including participants' understanding of their own medical status, fitness, and medication. The concept of having knowledge was interpreted as an understanding of their body helped participants to regain control over it. Participants commented they “Read the leaflet about what they do and what the side effects are” and asked, “Are they doing what they’re supposed to?”.

All participants were able to describe their medicines and medical treatments. In addition, they showed curiosity to know about their treatment, either finding answers themselves or using the knowledge of others. Gaye articulated her lack of knowledge about her medicines.

All the participants could recall the names, timings and strengths of their medicines, often referring to paper copies of medical notes during their interview, although these were not requested by the researcher. They found information through hospital consultants, GPs, acquaintances, family, support group and rehab group members. They used sources such as medicine information leaflets and academic-level databases. Two participants sought the knowledge of a private consultant, reasoning that the consultants’ professional status conferred a better level of care. One participant linked private healthcare with receiving better care and economic status.

### Considering the future

This superordinate theme was drawn out by participants as they invoked the idea of the future in all their narratives. This theme was interpreted as constructing certainty and reassurance. The uncertainty of the future illustrated by “not knowing where I was heading”. Gaye, Beki and Colin all mentioned taking medicines continuously into the future, using an almost identical phraseology of “having to take these forever”, and Beki’s “every day for the rest of my life”.

Gaye mentioned that the future is unknown in terms of heart health, and Beki considered that another heart attack is possible. Colin used the future conditional tense as he talked about having taken his medicine for such a long time “it had built up inside”, and that if he missed a dose, it “shouldn’t be a problem”.

### Assimilation into lifestyle

Another broad superordinate theme was assimilation of medication into the participants’ lives. All participants relayed how they accommodate their medicines into their everyday routine in a unique way. This group included themes of personal relationships, medicine location, systems and adaptations. This theme was interpreted as fitting medicine-taking to their life-world, a means of control and stability. They used phrases such as “Take them when it fits me”, and “It’s just a routine now”.

Gaye and Colin discussed being overwhelmed initially, but then how taking medication has become part of their everyday life. They both described the routine they had created, now as unremarkable and not noticeable. Both Beki and Chris mentioned a difficulty, Beki because of the side-effect, and Chris because of the extra quantity to remember, but they persisted with their set treatment.

Gaye began with swallowing difficulties but was given a “tip” by a pharmacist, and now takes medications simultaneously with a grape.

The location of medication was discussed as a practical decision or based upon their beliefs. Beki sites her medication in her kitchen, “out of the way” as her son’s safety is a priority. Chris decants all packaging and keeps them mixed in a Tupperware box, as an efficiency measure. Colin keeps his medication in a pouch which remains in one location at home, his GTN spray is the only item that he will carry in his pocket whilst at the gym.

Beki discussed how her pregnancy took priority over her medicine taking, as she stopped taking some of her medicines.

Chris and Colin talked about medicine-taking as an effortless activity, both using the analogy of coffee and tea-drinking to describe the automatic nature of taking medicines. Chris compared cultural drinking to taking different medicines, “coffee for that, wine for this”.

Neither Chris and Colin were concerned about missing doses, Chris reasoning that missing medicines wasn’t life-threatening, and Colin because he doesn’t notice any difference.

### Disturbance to routine

All the participants discussed how a disturbance to their daily routine could result in their missing a dose of medication. Disturbances included being away from home, being on holiday, and distraction by another activity. In opposition to the previous theme, this was interpreted as a destabilisation of their habitual activity. They described “The routine is a bit different” and saying, “I went to bed and forgot that I'd not taken my pills”.

Being away from home was discussed as a reason by all participants. Gaye talked about going out and forgetting her evening dose of statin. Beki suggested that she might be out and forget to take a dose. Chris took his medicine box out with him to ensure he did not miss doses.

Colin’s wife gave him verbal reminders when away on holiday and he mentioned different time zones causing confusion.

Chris gave the example of being absorbed on the internet and so went to bed without taking the evening dose. Colin discussed missing doses when his family came to stay at his home.

## Discussion

This study has produced themes that add to our understanding of the experience of medicine taking following a myocardial infarction. The participants discussed medicine taking through four superordinate themes. Firstly, they *compared themselves* to others, often using this comparison to bolster their sense of wellbeing and provide optimism about themselves. Secondly, *knowledge* was important to them, to help them construct an understanding of their condition and its management. Thirdly, the participants all considered the *future* in some form, thinking of it as either a constant or an unknown concept. Fourthly, participants discussed ways that they fitted their *medicines into their lifestyle* each making unique adaptations to manage their individual medicines. A related theme to lifestyle, all participants discussed how *a change to their routine* adversely affected their medicine taking. The study offers an interpretivist perspective in an area weighted towards quantitative research. The themes provide patients’ perspectives on medication, extending beyond the simple representation of adherence as either intentional or unintentional, helping therefore to give context to people’s engagement with medication following a myocardial infarction. These themes could help guide practitioners to provide more patient-centred care in future health consultations. They also show that patients adapt medicine taking into their lives in various and unique ways and a tailored approach to support them would be appropriate.

Currently medicine adherence support is provided to this patient group at the primary healthcare level through GP services, where the main aim is to integrate hospital discharge and return to independent home living through a programme of cardiac rehabilitation. While social support and cardiac rehabilitation have been well documented to improve treatment outcomes and quality of life measures, it is also apparent that recovery is a complicated and multifactorial phenomenon. An important positioning paper by the European Association of Cardiovascular Prevention and Rehabilitation highlights the complex role that psychosocial-related factors play in both the genesis and recovery of CHD^27^. For example, psychosocial factors such as stress, anxiety and depression are implicated in the development of CHD, can be caused by CHD, and even reduced by CHD—the latter occurring when family rally round to increase social support following a diagnosis. The paper also discusses the complex ways that psychosocial factors influence cardiac outcomes and recovery, and how these factors can overlap and work in multiple directions, for example acting as barriers to lifestyle changes and treatment adherence. This complexity is in line with the themes untangled in this analysis, some of which conceptually overlap and affect one another, further emphasising the need for a patient-centred approach.

The theme of comparison to others and seeing one’s own health protective behaviours as superior to others, could be construed as participants’ way of evidencing their own health, maintaining control, and/or providing self-reassurance. This theme of comparison to others was also discussed in a narrative analysis of patients taking anticoagulant medicines to prevent stroke^28^. In that study, participants also constructed themselves as superior to others, evidencing their good intentions for example as good adherers while non-adherers were ‘wasteful’ or ‘ungrateful’. Although a study of a different patient group, it agrees with this study that participants were keen to portray themselves as being healthy, adhering to medicines and positive lifestyle measures, and seeing others as having less favourable circumstances in terms of health. They found that missing doses was described as due to external circumstances of timing and location, and not linked to a personal trait. This supports the notion that participants are constructing an identity of positive health and higher morals than others to find comfort and gain control away from the tension of the uncertain. This finding highlights the need to consider the individual within group activities such as cardiac rehabilitation and social support groups, or when discussing benefits of treatment in terms of ‘most patients’.

In the current study, the theme of knowledge is important because of its role in creating coherence whereby a clear model of treatment helps participants remove uncertainty and regain control of their self over their illness. Another study of the meaning of medication to patients also explored the role that knowledge and meaning play in helping patients understand and manage their medication^29^. That study found the medication experience to be a meaningful encounter, with positive or negative bodily effects, and an unremitting nature which could cause patients to question the need for it. The patients could even exert control over the medication through the expertise gained from taking it (e.g., take the medication only ‘when required’). Parallels can be drawn with the current study under the themes of both knowledge and assimilation into lifestyle, as participants described their own experience of medication effects and side effects, sometimes questioning the need to take medication, and importantly, gaining control over the medication by simply taking it and not feeling it was unwarranted. This emphasis on meaning-making could be thought of as a learning process and therefore prompt practitioners to think about where a patient is in their learning journey when delivering patient-centred care.

Similarly, the theme of thinking about the future could add to a sense of coherence and continuity. It is noteworthy that medicines adherence is not often framed in relation to the concept of the future. In pharmacy, the professional focus is traditionally upon medical history and the future extends only as far as the patient’s current valid prescription. The use of electronic transfer of record keeping, sharing, prescribing, and repeat batches of prescriptions, however, is potentially of benefit in terms of framing the future. This suggests more work could be done to investigate the impact of the current model of monthly prescriptions on patients’ sense of their future and in turn their medication adherence.

Medicine-taking exists within the constructed life-world of the patient, affects and is affected by it. The theme of fitting into lifestyle, drawn out in this study, highlights the interconnectedness of medicines and patients’ life-world. In this study patients had incorporated their medicines into their lives in unique and specific ways that were important to them and their beliefs. This adds further weight to the argument that reducing medicine-taking to ‘cause and effect’ is over-simplistic and of limited practical use. Against the good adherence work that the participants discussed, missing doses was attributed to being distracted, away from home, and timing issues. This is similar to the findings of a qualitative interview study of unintentional non-adherers taking medication for chronic conditions^30^, which found schedule change, life pressures, and location change to be reasons for adherence failure, suggesting that work to improve adherence should focus on these routine-related factors. The theme of ‘change to routine’ is arguably not disease-specific but associated with medication taking in chronic illness more generally. Routine is rooted in physicality, related to time, space and occupation, which could all be manipulated through behaviour change therapies in order to remove practical and perceived barriers to taking medicines^31^. However, this type of intervention might only be relevant where patients are unintentional non-adherers, who aim to be good adherers (compared to others), rather than for intentional non-adherers who have no intention of taking medicines to start with.

A rich, detailed description of the lived experience of medication taking was achieved by the devotion of time, care and attention to the analysis. By using a case study approach, the results cannot claim to be exhaustive as there is no endpoint at data saturation as found in some qualitative methodologies. However, the use of comparison between cases has elicited superordinate themes for further investigation. In line with the ideographic nature of an IPA study, the results are not generalisable to larger populations but could be cautiously broadened to establish how they fit amongst different groups. IPA requires a homogeneity of sample with respect to the phenomena under investigation, and this was achieved through purposive selection of participants as all having experience of taking medication to prevent further AMI. This sample was similar due to their locality to the South-East of England, similar socio-economic profile and using UK NHS services to provide their healthcare. The results are not intended to be generalisable to other AMI populations outside of this specific context, but this is not the intention of IPA, instead finding universal patterns within specific detailed accounts drawn out by the analysis and thematic grouping work towards cautious claims of transferability^32^.

An equal gender mix was observed, but results were not de-aggregated, contrary to SAGER guidelines^33^. The rationale for this was the small participant number and ideography of an IPA methodology, where the focus is individual voice, and summary of detailed themes resonating through those voices, as opposed to a broader aggregation of themes.

The self-selection bias of participants may have affected the result of this study, as perhaps volunteers tend towards individuals with positive or more extreme experiences. The situational pressure of an interview may have contributed to the participants feeling obliged to satisfy the researcher’s line of questioning and present themselves in the best possible light, in the same way that a medical consultation might do^34^. The method of analysis, IPA, is a close first-person study, and so the participant is interpreted as being truthful and without agenda. Future study to examine the performance and politics of the talk during the same interviews could be completed using discourse or conversation analysis as a more critical method.

The use of IPA fitted well with the research question and compliments the model of patient-centred care where empathy and understanding are valued. This study contributes to the discussion of methodology in phenomenological studies as the integration of existential categories was found to be useful in the organisation and analysis of the data. Other studies have found an integrated phenomenological approach to be beneficial to the production of their findings, such as using similar lifeworld categories^35^, enactivism^36^ and use of a revised grounded methodology^37^.

This study has shown that whilst generalisations can be made, each patient’s experience is unique and the meaning they associate to their behaviour and action is very personal. Therefore, the case study approach to developing medicine-taking interventions is a valid one. This is congruent with NICE recommendations for medicine adherence support to be patient-centred with interventions adapted to individuals^12^, and future work to be directed towards this area. This patient-centredness could be practised in pharmacy by way of motivational interviewing as an aid to medicine adherence, which in the UK is not currently a standard practice within the NHS.

Whilst fulfilling the aim of describing the medicine-taking experience, the relationship to medication adherence, and more importantly, *non*-adherence is unclear. All the patients discussed rarely missing their doses, and so perhaps future studies could solely include the elusive volunteer group of non-adherent patients. This echoes the findings of the Cochrane review of adherence interventions^13^, which concluded that more work ought to be completed with patients whose adherence is low.

## Conclusion

This study found the meaning of medicine-taking in this participant group was oriented towards reducing the unknown and reinforcing stability and cohesion in their lives. This finding, alongside the themes that the analysis generated could help practitioners assisting patients in their medicine-taking experience. The theme of *comparison to others* was found to confer a moral superiority to the self and offers comfort against uncertainty. The theme of *knowledge* and looking *towards the future* contributed to a participant’s sense of coherence, again reducing anxiety of the unknown. *Assimilation into lifestyle* could be considered a modifiable learned behaviour, and *medication routines* could be strengthened by activity, location and timing interventions.

Meaning making was unique to individuals and so adherence interventions should be tailored to personal experiences in order to be more empathetic and therefore more impactful for an individual patient.

The methodology highlighted the importance of considering the phenomena of adherence as part of the whole life of an individual, as it is the entirety of a patient’s world that imparts meaning to adherence. The ideographic approach of this study produced a rich dataset and aligns with a tailored intervention to improving adherence and with a patient-centred approach.

## Supplementary Information

Supplementary Information.

## References

[1] Townsend N (2016). Cardiovascular disease in Europe: Epidemiological update 2016. Eur. Heart J..

[2] Chadwick Jayaraj, J., Davatyan, K., Subramanian, S. S. & Priya, J. Epidemiology of myocardial infarction. in *Myocardial Infarction*, vol. 54, 2083–2087 (IntechOpen, 2019).

[3] Bhatnagar P, Wickramasinghe K, Williams J, Rayner M, Townsend N (2015). The epidemiology of cardiovascular disease in the UK 2014. Heart.

[4] Jones, K., Saxon, L., Cunningham, W. & Adams, P. Secondary prevention for patients after a myocardial infarction: Summary of updated NICE guidance. *BMJ *(*Online*) **347**, f6544 (2013). 10.1136/bmj.f6544 24227827

[5] Smith SC (2011). AHA/ACCF secondary prevention and risk reduction therapy for patients with coronary and other atherosclerotic vascular disease: 2011 update. J. Am. Coll. Cardiol..

[6] Graham I (2007). European guidelines on cardiovascular disease prevention in clinical practice: Executive summary—Fourth Joint Task Force of the European Society of Cardiology and Other Societies on Cardiovascular Disease Prevention in Clinical Practice (Constituted by representatives of nine societies and by invited experts). Eur. Heart J..

[7] Ma, T.-T. *et al.* Effect of evidence-based therapy for secondary prevention of cardiovascular disease: Systematic review and meta-analysis. *PLoS One* **14**, e0210988 (2019). 10.1371/journal.pone.0210988 PMC6338367 30657781

[8] Nunes, V. *et al*. Medicines adherence. in *Medicines Adherence: Involving Patients in Decisions About Prescribed Medicines in Decisions About Prescribed Medicines and Supporting Adherence and Supporting Adherence* (, 2009). 21834197

[9] Naderi SH, Bestwick JP, Wald DS (2012). Adherence to drugs that prevent cardiovascular disease: Meta-analysis on 376,162 patients. Am. J. Med..

[10] Baroletti S, Dell’Orfano H (2010). Medication adherence in cardiovascular disease. Circulation.

[11] Sabaté E. eds. *Adherence to Long-Term Therapies: Evidence for action*. World Health Organization. (2003).

[12] *Medicines adherence: Involving patients in decisions about prescribed medicines and supporting adherence Clinical guideline* (2009). 21834197

[13] Nieuwlaat, R. *et al.* Interventions for enhancing medication adherence. *Cochrane Database Syst. Rev.* CD000011 (2014). 10.1002/14651858.CD000011.pub4 10.1002/14651858.CD000011.pub4 PMC7263418 25412402

[14] Van Dulmen S (2007). Patient adherence to medical treatment: A review of reviews. BMC Health Serv. Res..

[15] Abraham C, Kelly MP, West R, Michie S (2009). The UK national institute for health and clinical excellence public health guidance on behaviour change: A brief introduction. Psychol. Health Med..

[16] Vermeire, E., Hearnshaw, H., Van Royen, P. & Denekens, J. Patient adherence to treatment: Three decades of research. A comprehensive review. *J. Clin. Pharm. Ther.* **26**, 331–342 (2001). 10.1046/j.1365-2710.2001.00363.x 11679023

[17] Smith, F. Health services research methods in pharmacy: Qualitative interviews. *Int. J. Pharm. Pract.* **6**, 97–108 (1998).

[18] Rosenfeld D, Weinberg D (2012). Domestic practice, situated contingency and adherence to medical directives: A call for research. Soc. Theory Health.

[19] Pietkiewicz I, Smith JA (2012). A practical guide to using Interpretative Phenomenological Analysis in qualitative research psychology (Praktyczny przewodnik interpretacyjnej analizy fenomenologicznej w badaniach jakościowych w psychologii). Czas. Psychol..

[20] Zahavi D (2019). Getting it quite wrong: Van Manen and Smith on phenomenology. Qual. Health Res..

[21] Van Manen, M. *Phenomenology of practice*:* Meaning-giving methods in phenomenological research and writing*. (Routledge, 2016)

[22] Tong A, Sainsbury P, Craig J (2007). Consolidated criteria for reporting qualitative research (COREQ): A 32-item checklist for interviews and focus groups. Int. J. Qual. Health Care.

[23] Smith, J. A., Flowers, P. & Larkin, M. *Interpretative Phenomenological Analysis Theory, Method and Research*. (SAGE Publications Ltd, 2009).

[24] Rathbone, A. Durham E-Theses A phenomenological investigation of patients’ lived experiences of medicines adherence: A novel perspective for future intervention development.

[25] Kallio H, Pietilä A-M, Johnson M, Kangasniemi M (2016). Systematic methodological review: Developing a framework for a qualitative semi-structured interview guide. J. Adv. Nurs..

[26] Prescott, F. J. Validating a long qualitative interview schedule. *WoPaLP* **5** (2011).

[27] Pogosova, N. *et al.* Psychosocial aspects in cardiac rehabilitation: From theory to practice. A position paper from the cardiac rehabilitation section of the European association of cardiovascular prevention and rehabilitation of the European Society of Cardiology. *Eur. J. Prev. Cardiol.* **22**, 1290–1306 (2015). 28294802

[28] Hawking MKD, Robson J, Taylor SJC, Swinglehurst D (2020). Adherence and the moral construction of the self: A narrative analysis of anticoagulant medication. Qual. Health Res..

[29] Shoemaker, S. J. & Ramalho De Oliveira, D. Understanding the meaning of medications for patients: The medication experience. *Pharm. World Sci.* **30**, 86–91 (2008). 10.1007/s11096-007-9148-5 PMC2082655 17653833

[30] Huyard C, Haak H, Derijks L, Lieverse L (2019). When patients’ invisible work becomes visible: Non-adherence and the routine task of pill-taking. Sociol. Health Illn..

[31] Horne R, Cooper V, Wileman V, Chan A (2019). Supporting adherence to medicines for long-term conditions. Eur. Psychol..

[32] Smith JA (2004). Reflecting on the development of interpretative phenomenological analysis and its contribution to qualitative research in psychology. Qual. Res. Psychol..

[33] Heidari S, Babor TF, De Castro P, Tort S, Curno M (2016). Sex and gender equity in research: Rationale for the SAGER guidelines and recommended use. Res. Integr. Peer Rev..

[34] Anyan, F. *The Influence of Power Shifts in Data Collection and Analysis Stages: A Focus on Qualitative Research Interview*. *The Qualitative Report* **18** (2013).

[35] Van Reenen, E., Van Der Borg, W., Visse, M., Van Der Meide, H. & Visser, L. Fear, fight, familiarize: The experiences of people living with relapsing-remitting multiple sclerosis and taking oral medication. *Int. J. Qual. Stud. Health Well-being* **14** (2019). 10.1080/17482631.2019.1648946 PMC6713094 31390951

[36] Stilwell, P. & Harman, K. Phenomenological research needs to be renewed: Time to integrate enactivism as a flexible resource. *Int. J. Qual. Methods* **20** (2021).

[37] Køster, A. & Fernandez, A. V. Investigating modes of being in the world: An introduction to Phenomenologically grounded qualitative research. *Phenomenol. Cogn. Sci.* 1–21 (2021). 10.1007/s11097-020-09723-w

