# Supplementary material for: A phenomenological analysis of the experience of taking medication to prevent a further heart attack
Source: Sci Rep. 2021 Dec 6;11:23468. doi: 10.1038/s41598-021-02909-5 (PMC8648863; doi:10.1038/s41598-021-02909-5)
Supplement: Supplementary file 1 — Supplementary Information. [file 41598_2021_2909_MOESM1_ESM.pdf]

University of Reading

Interview Schedule

- Go over information sheet verbally, opportunity to answer questions
- Sign consent form and give copy to participant.

Thanks for agreeing to take part today.

The interview is entirely your space to tell me about your experiences of the medicines that you have been given for your heart.

There are no right or wrong answers, and everything that you say is of value to us.

I'm going to ask you a question, and I'd like you have a think, then give me as much detail in your answer as you can.

Take as long as you need and include as much as you can think of in your reply.

If there are any questions that you don't want to answer, or you want to move on, we'll do that.

You may find that I'm asking you about things that might seem really obvious, but I want to get right down to the details of the things that you say. As though I am totally new here and am trying to figure out what's going on and how things work.

At the end, I'm going to ask if there are any topics that you feel I may have missed in my questions, so if you could have a think about that as we go, also.

Any questions? Are you OK to start?

- Turn on recorder

1) We're researching with people who've had a heart attack- How long ago did you have yours?

2) Could you tell me more about what happened?

3) Could you describe how you were introduced to your new medicines?

4) Can you describe what it felt like to be given new medicines?

5) Can you tell me the names and a bit about the medicines that you take?

6) Could you tell me your daily medicine routine?

7) How does that fit within your life?

8) Could you explain what it feels like when you take your heart medicine?

9) Is it the same feeling when you take other types of medicines?

10) Do you have anything that helps you to take them?

11) Can you think back to a time recently that you didn't take them. Could you describe what happened?

12) After missing your medicine, what did you feel?

13) On a scale of your priorities in life, where would you put taking your medicines?

14) Do you feel rewarded by taking them, if not, what do you feel?

15) What types of feelings go through your mind in relation to taking your medicines?

16) How much do you feel that taking your medicine plays a part in keeping you healthy?

17) What other things are important in terms of keeping healthy?

18) Do you know anyone else in a similar situation as you?

19) Do you see the hospital staff often? Are they useful, and in what way?

20) Is there anyone helpful to you that you see in your surgery?

21) And in the pharmacy?

The next section of questions is about your feelings about the medicine you take. Take all the thinking time you need.

22) What is your view of the medicines that you take for your heart?

23) Do you consider them (your heart medicines) different to other types of medicines?

Last couple of questions-

24) How would you describe your relationship with medicines?

25) Do you think that it's changed since having a heart attack?

And lastly.

26) With a view to medicine taking do you think that there are any questions that I should be asking people about which we haven't covered yet?

Thank you. All done.

I'm going to give you a different name and use that to write up the interview. I will email you a copy and let me know if there's anything you want to change or disagree with.

I'll keep in touch, and let you know how the study is going, but it may be a year or two before you hear.

Participants with cause for concern:

> You mentioned that… Perhaps if you talked about this with a pharmacist or someone in the surgery trained to help, I'm sure that there are ways that they can help you to sort out the issues that you're having.

Or further:

> Let me put you in touch with somebody, which pharmacy do you go to?
